# A method for microplastic extraction from ornithogenic soils

**DOI:** 10.1016/j.mex.2026.104031

**Published:** 2026-07-04

**Authors:** Megan Reaves, Helena Ruffell, Tanya O’Neill, Sally Gaw

**Affiliations:** a School of Physical and Chemical Sciences, University of Canterbury, Christchurch, New Zealand; b School of Science, University of Waikato, Hamilton, New Zealand

**Keywords:** Ornithogenic soils, Microplastics, Extraction methods, Digestion, μ-FTIR

## Abstract

Organic rich samples require digestion prior to extracting microplastics for spectroscopic analyses. However, it can be challenging to fully digest samples containing high amounts and multiple types of organic matter without causing degradation or complete loss of the targeted polymers. Ornithogenic soils contain algae, feathers, chitinous krill, and lipid rich guano which may be difficult to fully break down using single-step digestion methods. We investigated suitable methods for microplastic extraction from ornithogenic soils by carrying out a combination of oxidative (Fenton’s reagent, H_2_O_2_, NaClO, and alkaline (10% KOH) digestion methods. The optimal method was a three-step digestion method using NaClO, 10% KOH, and 30% H_2_O_2_ at temperatures of ≤40 °C. The organic material present was sufficiently digested without comprising microplastic identification. Reference microplastic (100–5000 μm) recoveries ranged from 96 to 100% for spiked ornithogenic soils and 92–98% for procedural blanks. This three-step digestion method is recommended for organic rich guano and ornithogenic soil samples where microplastic extraction would not be possible otherwise.

• Organic matter in the ornithogenic soil samples was successfully digested using NaClO, 10% KOH, and 30% H_2_O_2_.

• Microplastic extractions were completed in five days.

## Graphical abstract

**Figure:**
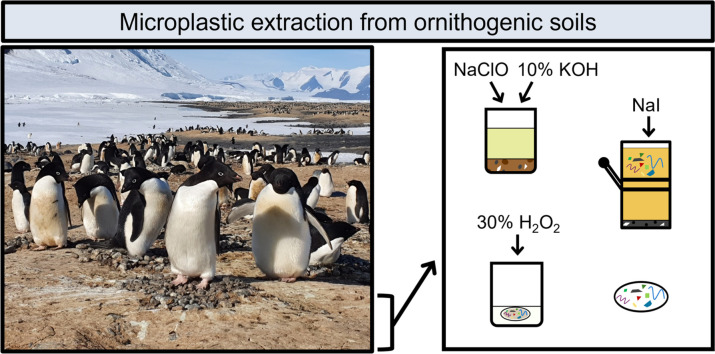

## Specifications table

**Table:** 

**Subject area**	Environmental Science
**More specific subject area**	Microplastic extraction
**Name of your method**	Microplastic extraction from organic rich ornithogenic soils by a multi-step digestion using potassium hydroxide, sodium hypochlorite, and hydrogen peroxide with a sodium iodide density separation
**Name and reference of original method**	M.B. Alfonso, K. Takashima, S. Yamaguchi, M. Tanaka, A. Isobe, Microplastics on plankton samples: Multiple digestion techniques assessment based on weight, size, and FTIR spectroscopy analyses, Marine Pollution Bulletin 173 (2021) 113,027. https://doi.org/10.1016/j.marpolbul.2021.113027. M.Z. Gouda, S. Roberge, L. Khiari, R. Benjannet, M. Desrosiers, Novel integrated workflow for microplastics extraction, quantification, and characterization in organic fertilizing residuals using micro-Fourier transform infrared spectroscopy (μ-FTIR), Chemosphere 377 (2025) 144,357. https://doi.org/10.1016/j.chemosphere.2025.144357. S.S. Monteiro, T. Rocha-Santos, J.C. Prata, A.C. Duarte, A.V. Girão, P. Lopes, T. Cristovão, J.P. Da Costa, A straightforward method for microplastic extraction from organic-rich freshwater samples, Science of The Total Environment 815 (2022) 152,941. https://doi.org/10.1016/j.scitotenv.2022.152941. J.C. Prata, J.P. Da Costa, A.V. Girão, I. Lopes, A.C. Duarte, T. Rocha-Santos, Identifying a quick and efficient method of removing organic matter without damaging microplastic samples, Science of The Total Environment 686 (2019) 131–139. https://doi.org/10.1016/j.scitotenv.2019.05.456. H. Ruffell, O. Pantos, B. Robinson, S. Gaw, A method for the extraction of microplastics from solid biowastes including biosolids, compost, and soil for analysis by µ-FTIR, MethodsX 12 (2024) 102,761. https://doi.org/10.1016/j.mex.2024.102761.
**Resource availability**	Aluminium foil Thermometer Glass stirring rod Crystallising dish (12 cm diameter) Ultra-pure water HPLC grade acetone 250 mL glass beakers 500 mL glass beakers 50 mL glass beakers Glass pipette Muffle furnace Hotplate Litmus paper Sodium hypochlorite (NaClO; 10–15% active chlorine) 10% Potassium hydroxide 30% Hydrogen peroxide Concentrated Hydrochloric acid (HCl; 36%) Sodium iodide solution (1.8 g cm^−3^) Glass vacuum filtration unit GF/C glass microfibre filters (1.2 μm pore, 47 mm diameter)

## Background

In recent years, quantifying the abundance of microplastics (< 5 mm) in environmental samples has become critical for understanding both the potential threats microplastic pollution pose to seabirds and the role seabirds such as penguins play in the accumulation of microplastics in coastal soils [[1], [2], [3], [4]]. Prior to spectroscopic analyses, microplastics are typically isolated and extracted from samples by a chemical digestion followed by a density-based separation. Simple single-step digestion and extraction methods may not be effective for organic rich matrices such as wastewater, soil, and sediments, which contain both high amounts and diverse types of organic matter. A wide variety of digestion and density separation methods have been used to isolate microplastics from organic rich matrices for subsequent analysis by Fourier Transform Infrared Spectroscopy (FTIR). The most widely used digestion methods for organic rich matrices involve wet peroxide oxidation (Fenton’s reagent, H_2_O_2_, NaClO), acid (HCl, HNO_3_), alkaline (KOH, NaOH), or enzymatic (Chitinase, Proteinase K) treatments [5]. Microplastics are then separated from the remaining sample by adding a salt solution, such as CaCl_2_, KI, NaI, NaCl, or ZnCl_2_, with a greater density than that of the targeted polymers [5]. Ornithogenic soils, including penguin influenced soils, can contain high amounts and diverse types of organic material such as algae, feathers, chitinous krill, and lipid rich guano [6,7]. Consequently, single-step digestion methods may not be able to fully break down all organic matter, especially in samples from polar regions where the cold and dry climatic conditions preserve organic matter preservation.

Multi-step digestions have been developed for guano and ornithogenic soil samples using a combination of KOH, H_2_O_2_, and HNO_3_ based treatments [[8], [9], [10]]. However, those applied to guano may not be effective at breaking down all materials in ornithogenic soils unless the materials are removed manually or through sieving which can increase risk of exposure of the analyst to airborne pathogens or result in cross contamination of the sample [8]. Robust digestion methods using acids or those with digest temperatures above 40 °C, while effective at breaking down the majority of organic material in ornithogenic soils, can lead to significant polymer degradation, polymer misidentification, or loss of polymers [5,[11], [12], [13], [14]].

**Fig. 1:**
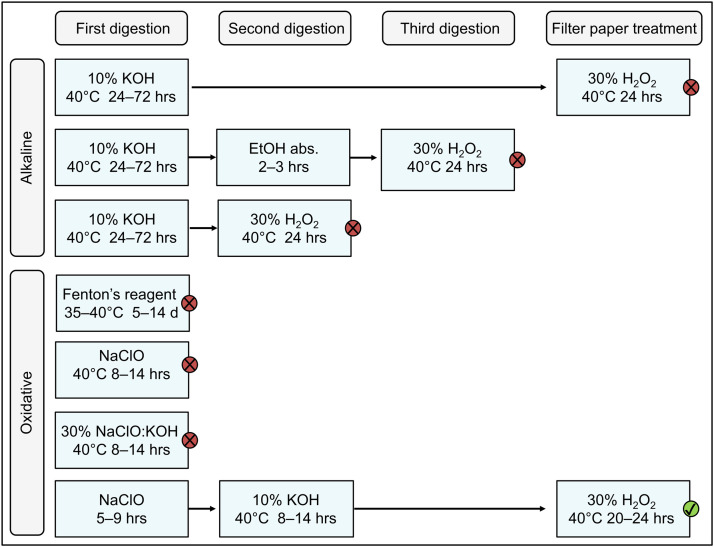
Working flow chart of the digestion methods tested. Ineffective methods are indicated by X shapes. The specific ineffective digestion step in each method is denoted by an asterisk (further detail in Appendix 1).

Herein published digestion methods were adapted to develop an efficient microplastic extraction method for ornithogenic soils to ensure both (i) the complete digestion of organic material such as feathers, chitin, lipids, and algae in ornithogenic soils; and (ii) ensuring polymers were preserved to the extent that they could be successfully identified using micro-Fourier transform infrared spectroscopy (μ-FTIR) with recoveries of spiked polymers ≥ 90%. A total of seven previously published digestion methods using a combination of solutions including Fenton’s reagent, 10% KOH, 30% H_2_O_2_, and NaClO (10–15% active chlorine) were adapted and trialled [[14], [15], [16], [17], [18], [19], [20], [21], [22]]. Method descriptions, schematics, and outcomes for methods that were found to be unsuitable for ornithogenic soil samples are presented in Appendix 1. Here a multi-step digestion method using 10–15% NaClO, 10% KOH, and 30% H_2_O_2_ was developed (Fig. 1).

## Method details

### Sample collection

Soil samples were collected from two active Adélie penguin colonies located at Cape Bird (77°10′S 166°41′E) and Cape Hallett (72°19′S 170°13′E), Antarctica, during the austral summers of 2022/2023 and 2023/2024, respectively. Samples were collected from soil pits (50 × 50 cm) using stainless steel trowels. Soils from Cape Bird were stored in 300 mL glass jars. Cape Hallett could only be accessed via helicopter and due to weight restrictions samples were stored in aluminium trays with an aluminium foil wrapped cardboard lid and were transferred to glass jars upon arrival. Soil samples were shipped to the University of Canterbury and stored at 3 °C until sample analysis.

### Chemicals and materials

The chemicals used for the digestion methods trialled were as follows: ultra-pure water (ASTM type I water, > 18.2 MΩ) sourced from a Sartorius Arium® pro filtration system fitted with a 0.45 μm filter (Sartorius AG), Decon90 detergent (Thermo Fisher Scientific New Zealand Ltd), HPLC grade acetone (Thermo Fisher Scientific Inc. Massachusetts, USA), concentrated hydrochloric acid (conc. HCl; 36%, Thermo Fisher Scientific Inc.), 30% hydrogen peroxide (H_2_O_2_) prepared using 35% H_2_O_2_ (ECP Ltd.), 10% KOH prepared with analytical reagent grade potassium hydroxide (KOH; Thermo Fisher Scientific), sodium hypochlorite (NaClO) with 10–15% active chlorine. Digestions followed a density separation step using sodium iodide (NaI) prepared using NaI salts (Livestock Supplies New Zealand).

### Quality assurance and quality control

Several precautions were taken to reduce airborne contamination during sample collection, processing, and analysis. Each test pit included one field blank to identify any contamination introduced during sample collection. Field blanks were prepared by placing an empty open glass jar next to the test pit during sample collection. Once in the laboratory, field blanks were rinsed with ultra-pure water over a vacuum filtration unit and filtered through Whatman grade GF/C glass microfibre filters (1.2 μm pore, 47 mm diameter), hereinafter referred to as filter papers. Cotton lab coats were worn during all laboratory procedures and analysis. All metal laboratory equipment and glassware used were washed with Decon90 detergent followed by three rinses in ultra-pure water, one rinse in HPLC grade acetone, and immediately wrapped or sealed with aluminium foil to reduce the risk of airborne particulate contamination. Laboratory procedures including solution preparation was carried out in fume hoods cleaned with 70% ethanol and lined with aluminium foil. Procedural blanks underwent the same laboratory methods as soil samples to identify any background contamination. Particles identified in the soil samples with the same characteristics as particles found in the field or procedural blanks were omitted from the results. Prior to sample filtration, the filter papers were baked at 450 °C in a muffle furnace for four hours to ensure any polymers introduced from the packaging were destroyed. The NaI, 30% H_2_O_2_, and NaClO solutions were filtered prior to use [23]. The microplastic extraction method, including digestion, density separation, and filter paper treatment steps, was able to be completed in five days, thereby reducing the processing time and the associated risks of contamination.

### Sample preparation

The final microplastic abundance was reported in particles per gram of soil (dw). For each sample, 5 g of soil was weighed directly into a 500 mL glass beaker and immediately covered with aluminium foil. To minimise handling, the final particle count was converted from wet weight (ww) to dry weight (dw) by obtaining the moisture content from a separate 10 g subsample. The 10 g subsample was transferred to 50 mL falcon tube and placed in a freeze drier (Thermo Savant SuperModulyo (Thermo Fisher Scientific)) for 48 h.

### Method validation

For method validation, procedural blanks (n = 3) and ornithogenic soil samples (n = 3) were spiked with reference polymers (Appendix 2). Three different ornithogenic soils were spiked to determine the reproducibility of the method. The reference polymer fragments (500–1000 μm; 1000–5000 μm) were purchased from Clariant (high-density polyethylene (HDPE), high impact polystyrene (HIPS), polyamide (PA), polyethylene terephthalate (PET), and polypropylene (PP)), Chi Mei Corp. (acrylonitrile butadiene styrene (ABS)), and Marley (polyvinyl chloride (PVC)). The polyethylene (PE) microbeads (100–500 μm) were extracted from an over-the-counter facial cleanser and the polymethyl methacrylate (PMMA) fibres were prepared from yarn (1 mm) purchased at a craft store.

After the microplastic extraction methods were complete, the reference fragments and fibres on the filter papers were counted and visually inspected under a stereomicroscope (Nikon SMZ1270, magnification 12.7x). Reference polymers were transferred from filter papers to calcium fluoride discs (CaF_2_; 13 mm diameter x 0.55 mm) and analysed using µ-FTIR (Bruker HYPERION 2000 coupled to a Vertex 70 spectrometer and equipped with a liquid nitrogen cooled mercury cadmium telluride (MCT) detector and KBr beamsplitter) before and after extraction methods were carried out. Each particle was scanned using Opus software version 7.8. Particles were scanned 32 times in transmission mode at 15x magnification with a resolution of 4 cm^−1^ and a spectral range of 4000–1000 cm^−1^.

### Digestion methods

**Fig. 2:**
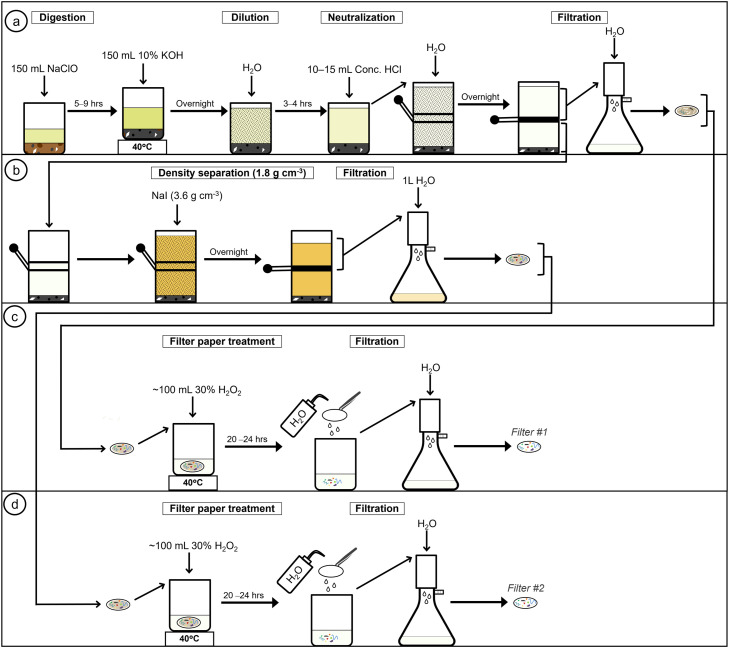
Schematic of the microplastic extraction method where a) soils were digested using NaClO (10–15% active chlorine) and 10% KOH, b) the remaining digest was subject to a NaI density separation, and c) and d) each filter paper from the digest and NaI density separation filtration steps were treated with 30% H_2_O_2_.

Each soil sample (5 g ww) was weighed directly into a 500 mL glass beaker and immediately covered with aluminium foil. The soils were treated with NaClO and 10% KOH as two distinct steps (Fig. 2). The NaClO was first used as an oxidising agent to remove soil organic matter at ambient temperature without heating the sample as would be required for other oxidising methods (e.g. Fenton’s reagent). The 10% KOH was used to remove any remaining feathers. Soils were first treated with 150 mL NaClO at room temperature. After 5–9 h, once gas bubbles had subsided, 150 mL of 10% KOH was added, and the aluminium foil covering the beaker was replaced by a glass crystalising dish to prevent the aluminium foil from corroding. The digest was then placed on a hotplate at 40 °C overnight. The following day, the digest was removed from the hotplate and cooled to room temperature. The digest was adjusted to pH 7 using 10–15 mL of conc. HCl. A neutralisation step was necessary to prevent the KOH from damaging the borosilicate GF/C filter papers and sediment microplastic isolation (SMI) units [24]. Digests were then transferred to a SMI unit where microplastic extraction and density separation methods described by Ruffell et al. [16] were carried out. Once in the SMI units, digests were made up to 650 mL with ultra-pure water and left to settle overnight. The following day the settled solids were separated from the supernatant by closing the valve and the supernatant containing low-density microplastics was decanted into a vacuum glass filtration unit containing a filter paper. The addition of 650 mL ultra-pure water to the SMI unit was necessary to prevent degradation of the SMI unit.

### Density separation method

The sample digestions were followed by a NaI density separation (1.8 g cm^−3^) to isolate microplastics. The SMI unit was opened and 340 mL of NaI (3.6 g cm^−3^), the volume equivalent to the volume of the bottom half of the unit, was added to the top half of the unit. The final density of NaI in the SMI unit was 1.8 g cm^−3^ upon mixing. The solids were then left to settle overnight. The following day the settled solids were closed off by turning the valve containing high-density microplastics (1.0–1.8 g cm^−3^) and decanted into a vacuum glass filtration unit containing a filter paper. In a final rinse step, NaI residues were removed from the filter paper by vacuum-filtering 1 L of ultra-pure water.

### Filter paper treatment

Both filter papers from the digestion and density separation contained chitinous material. The chitinous material was further digested by placing the filter papers in a 250 mL beaker containing ∼100 mL of 30% H_2_O_2_ and heating on a hot plate at 40 °C for 20–24 h. The filter papers were then removed from the solution using stainless steel forceps and rinsed with ultra-pure water. The remaining solution was filtered and contained little to no chitinous material.

## Method validation results

Recovery rates were 96%, 100%, 100% for spiked ornithogenic soil samples and 92%, 92%, 98% for spiked procedural blanks (Appendix 2). No major spectral changes to the reference polymers were identified (Appendix 3).

## Limitations

While protective of the polymers, maintaining the temperature of ≤40 °C can reduce sample digestion efficiency and unreasonably extend sample processing time. The multi-reagent method proposed herein provides a compromise for optimising digestion efficiency whilst protecting polymer integrity and took five days to complete compared to 10 days for a single reagent method [16].

The microplastic extraction methods developed here are compatible with μ-FTIR analysis which has additional limitations. Notably, μ-FTIR analysis requires visual identification of particles located on the GF/C glass microfibre filters. Particles of similar colour to the filter paper surface, specifically white or transparent particles, are less likely to be detected by the analyst [25]. While this may lead to an underestimation in white or transparent particles this may be the most suitable method when no other microplastic analysis (e.g., Pyrolysis-gas chromatography-mass spectrometry (Py-GC/MS), Laser Direct Infrared Spectroscopy (LDIR), or automated visual identification software) is feasible.

Additionally, as the reference polymers ranged from 100 to 1000 μm in size, the effectiveness of the digestion and extraction method was not validated for particles between 50 and 100 μm. Reference polymer ranging in size from 50 to 100 μm were not used for method validation as it can be challenging to ensure that particles below 100 μm in size have been added to each spiked sample successfully. Generally, for μ-FTIR analysis it is possible to identify particles as small as 50 μm however the success rate for transferring particles from the filter paper to the CaF_2_ disc for particles smaller than 100 μm is low [25]. For that reason, particles smaller than 100 μm in size are frequently omitted from μ-FTIR analysis. Therefore, we selected reference polymer fragments that were no smaller than 100 μm to represent common microparticle analysis practices.

## Conclusions

In conclusion, the proposed three-step microplastic extraction method, using NaClO, 10% KOH, and 30% H_2_O_2_ at temperatures of ≤40 °C, proved effective at digesting all organic matter present in ornithogenic soil samples (e.g., algae, feathers, chitinous krill, and lipid rich guano) without compromising microplastic identification or the recovery rates of all nine reference polymer types (100–5000 μm) tested. Additionally, this method was completed within five days, thereby reducing the risk of contamination and loss of particles. This method is recommended for organic rich guano and ornithogenic soil samples where microplastic extraction prior to FTIR analysis would not be possible otherwise.

## Ethics statements

Institutional ethical approval was not required as the direct handling of animals was not required for this study. Soil samples were collected under the appropriate permits. Sampling methods followed standard operating procedures approved by Antarctic New Zealand which minimise disturbance to nearby wildlife and overall environmental impact.

## CRediT authorship contribution statement

**Megan Reaves:** Conceptualization, Investigation, Methodology, Validation, Writing – original draft. **Helena Ruffell:** Methodology, Writing – review & editing. **Tanya O’Neill:** Funding acquisition, Project administration, Supervision, Writing – review & editing. **Sally Gaw:** Conceptualization, Funding acquisition, Project administration, Supervision, Writing – review & editing.

## Declaration of competing interest

There is no conflict of interest.

## Data Availability

All data is provided in the supplementary material.

## Appendix Supplementary materials

Image, application 1
