# Supplementary material for: A method for microplastic extraction from ornithogenic soils
Source: MethodsX. 2026 Jul 4;17:104031. doi: 10.1016/j.mex.2026.104031 (PMC13400951; doi:10.1016/j.mex.2026.104031)
Supplement: Supplementary file 1 [file mmc1.docx]

**Supplementary material**

A method for microplastic extraction from ornithogenic soils

Megan Reaves^a*^, Helena Ruffell^a^, Tanya O’Neill^b^, Sally Gaw^a^

^a^School of Physical and Chemical Sciences, University of Canterbury, Christchurch, New Zealand; ^b^School of Science, University of Waikato, Hamilton, New Zealand

Appendix 1. Descriptions of methods trialled

Overall, seven digestion methods were trialled using Fenton’s reagent (method #1), 10% KOH (method #2), 10% KOH followed by ethanol absolute (method #3),10% KOH followed by 30% H_2_O_2_ (method #4), NaClO (method #5), 1:1 ratio of NaClO:KOH (method #6), and NaClO followed by the addition of 10% KOH (method #7). Methods #1–6 were not suitable for ornithogenic soil samples. Descriptions of Methods #1–6, including the outcomes, are provided below.

*Fenton’s reagent*

For method #1, we applied digestion methods using Fenton’s reagent as described by Hurley et al. [1] and modified by Ruffell et al. [2]. Soils were digested using Fenton’s reagent where 50 mL of 0.05 M Fe (II) solution (7.5 g of iron sulfate heptahydrate (Thermo fisher Scientific Inc.), 3 mL of concentrated sulfuric acid, and 500 mL of ultra-pure water), 50 mL ultra-pure water, and 10–50 mL of 30% H_2_O_2_ was added to the sample (Fig. S1). The volume of 30% H_2_O_2_ and ultra-pure water varied depending on soil reactivity. Generally, 30% H_2_O_2_ was added in 10–20 mL increments once per day for 3–5 days and once the initial digestion reaction was minimal the digest was placed on a hotplate at 35–40°C. This step was repeated for up to two weeks until the digestion was complete. The digest was complete once all solids sunk to the bottom of the beaker, the solution was opaque, and no bubbles or fizzing were observed. This method was successful at fully digesting non-ornithogenic soil samples and ornithogenic soil containing moderate amounts of organic matter within the first week. It was not always possible to complete a full digestion on the ornithogenic soil samples with high amounts of organic matter. Soil samples with high organic matter were highly reactive and were susceptible to bubbling over each time 5–10 mL of 30% H_2_O_2_ was added, especially during the first week of digestion. In some cases, the production of bubbles did not occur in soil samples with high organic matter for up to two hours after the first 10 mL aliquot of 30% H_2_O_2_ was added.

Stirring the solution with a glass stirring rod did not reduce the bubbles effectively and therefore ultra-pure water was added to reduce the bubbles. The bubbles produced often lifted the soil material to the top of the beaker and if bubbles subsided undigested soil material adhered to the walls of the beaker. Ultra-pure water was then added to reincorporate the undigested soils back into the solution. Consequently, the solution volume needed to successfully digest the sample exceeded that of the beaker.


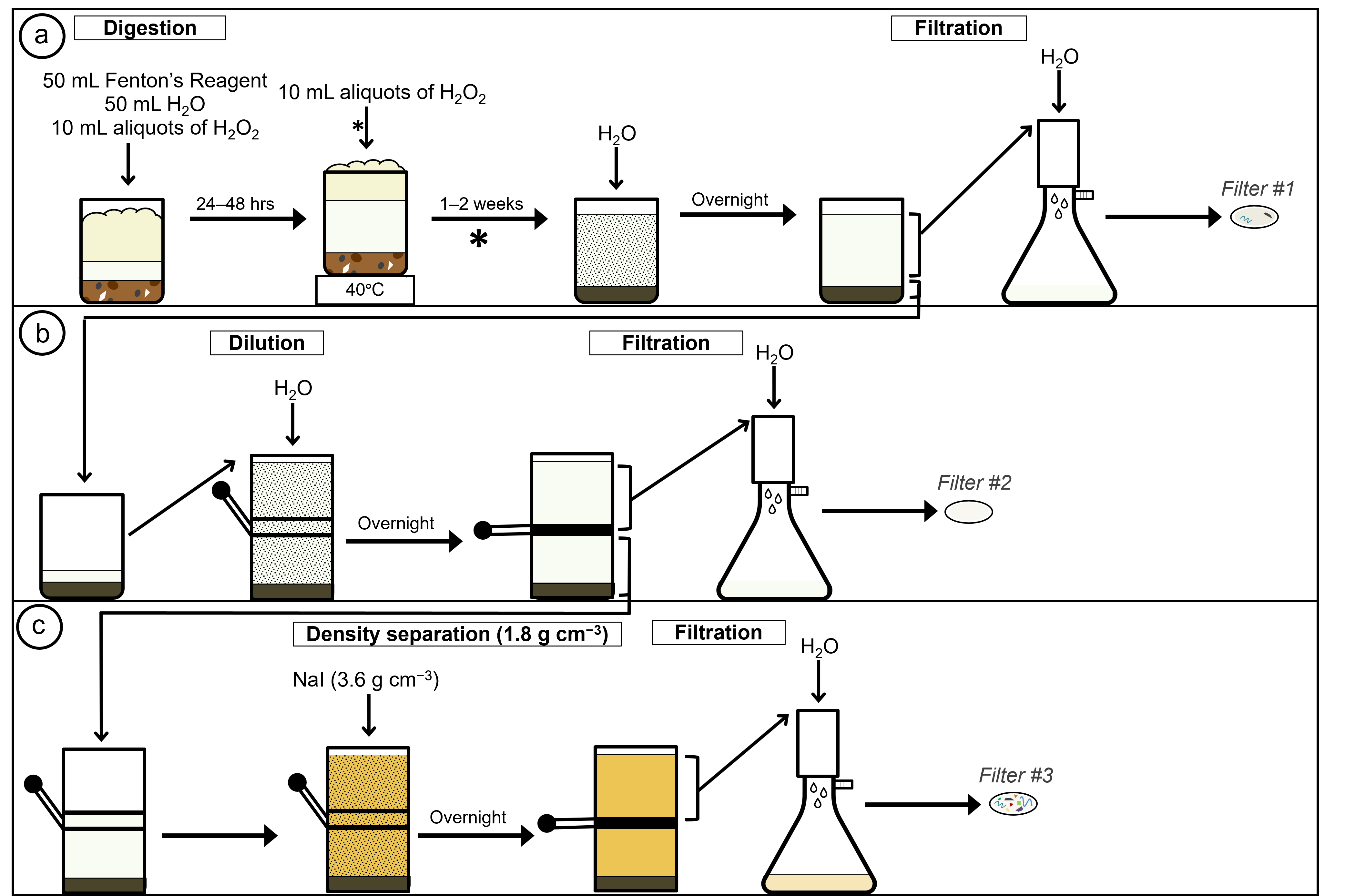


Figure S1. Schematic of method #1 where a) soils were digested using Fenton’s reagent, diluted, and filtered, b) the dilution and filtration steps were repeated, and c) the remaining digests were subject to a NaI density separation.

*Alkaline digestions using 10% KOH*

Microplastics have been successfully extracted from freshly deposited penguin guano using a 10% KOH digestion and subsequent treatment of the filter paper with 10% H_2_O_2_ at 40°C for 24 hours [3]. Treatments involving 10% KOH at 50°C for 6 hours have been effective at digesting both feathers and fish tissues [4].

We investigated the efficacy alkaline digestions using 10% KOH (Method #2), 10% KOH followed by ethanol absolute (EtOH; Method #3), and 10% KOH followed by 30% H_2_O_2_ (Method #4). We reduced the temperature to 40°C as treatments with 10% KOH at temperatures above 50°C may cause damage to some polymers [5]. Due to the high chitin content from krill, we used 30% H_2_O_2_ which has been previously used to remove chitin from plankton samples [6]. As KOH can degrade aluminium foil, all digests containing KOH were covered with glass crystalising dishes.

For the first alkaline digestion, method #2, ornithogenic soil samples were treated with 100 mL of 10% KOH and then placed on a hotplate at 40°C for 24–72 hours (Fig. S2). Immediately after the addition of 10% KOH the solution turned from a clear liquid to a dark brown solution making it difficult to determine if organic matter within the solution had been fully digested. After the initial digestion, the solution was made up to 500 mL with ultra-pure water and left to settle overnight. The following day the top of the solution was filtered. However, filter papers would often clog for ornithogenic soil samples with high lipid contents. Samples with high lipid contents can undergo saponification forming a thick gel layer in the presence of KOH [7]. Up to three filter papers were required to filter the upper 300–400 mL of the solution and in some samples, partially digested feather shafts were resuspended into solution following the dissolution step.

**
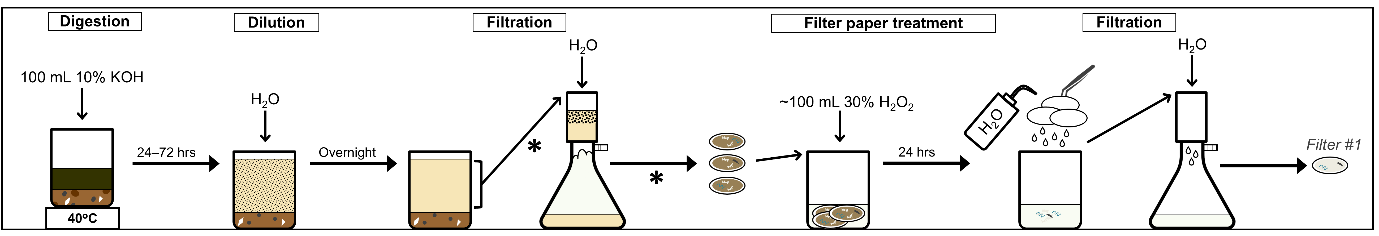
**

Figure S2. Schematic of method #2 where soils were digested using 10% KOH and filter papers were treated with 30% H_2_O_2_.

Treating a 10% KOH digest with EtOH has been demonstrated to be successful at redissolving saponified samples [7]. For method #3, an additional step was introduced involving a 1:4 EtOH (Thermo Fisher Scientific), treatment, in which 25–50 mL of EtOH was added to each sample following the initial digestion in 10% KOH at 40°C for 24–72 hours (Fig. S3). After 2–3 hours the solution was made up to 500 mL with ultra-pure water and left to settle overnight. The following day the top of the solution was successfully filtered and there was no gel layer present on the filter paper. An additional dilution step was carried out to prevent a reaction between any remaining KOH and NaI. The remaining digest was made up to 500 mL with ultra-pure water, left to settle overnight, and the top of the solution was filtered the following day. The dilution step was repeated 2–3 times until the pH of the filtrate was neutral. The remaining density separation and H_2_O_2_ filter paper treatment steps were successful however, feather shafts were still present in the sample.


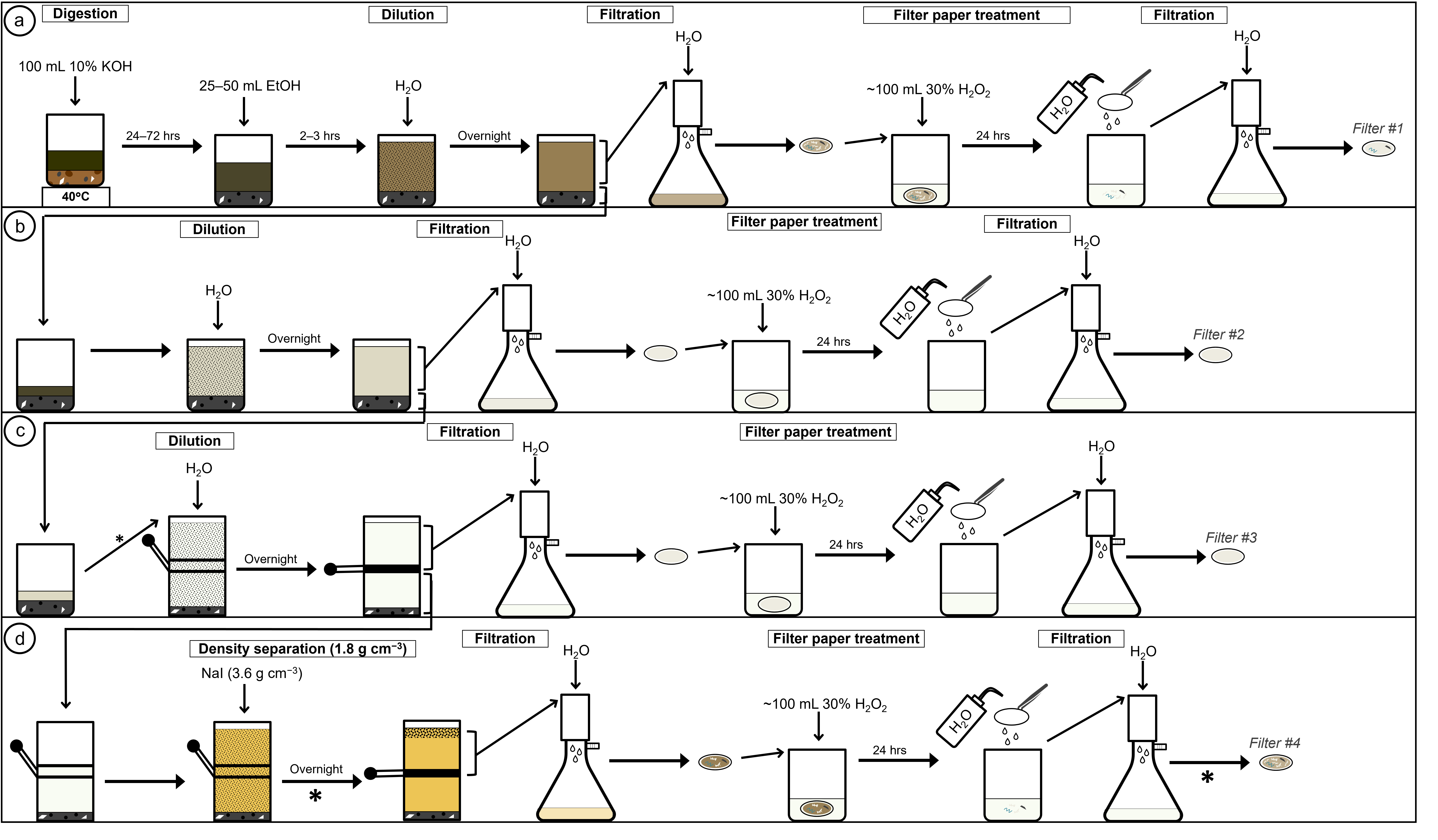


Figure S3. Schematic of method #3 where a) soils were digested using 10% KOH and EtOH, b) digests were diluted with ultra-pure water, filtered, and the filter papers were treated with 30% H_2_O_2_, c) dilution steps were repeated, and d) the remaining digest were subject to a NaI density separation, filtered, and the filter papers were treated in 30% H_2_O_2_.

For the last alkaline digestion, method #4, the EtOH treatment and dilution steps were replaced with a neutralising step using concentrated HCl followed by a further digestion in 30% H_2_O_2_ (Fig. S4). More specifically, after the initial 10% KOH digestion step, the digest was brought to room temperature, and up to 10–15 mL of HCl was added slowly until the pH was neutral. The digest was then placed on a hotplate at 40°C and 10–15 mL of 30% H_2_O_2_ was gradually added. The main advantage of treating the digest directly with 30% H_2_O_2_ was that filter papers no longer needed an additional 30% H_2_O_2_ treatment. After 24 hours the digest was removed from the hotplate and made up to 500 mL with ultra-pure water. The top portion of the digest was then filtered the following day. While the additional digest treatment using 30% H_2_O_2_ prevented saponification, excess organic matter would often clog filter papers.


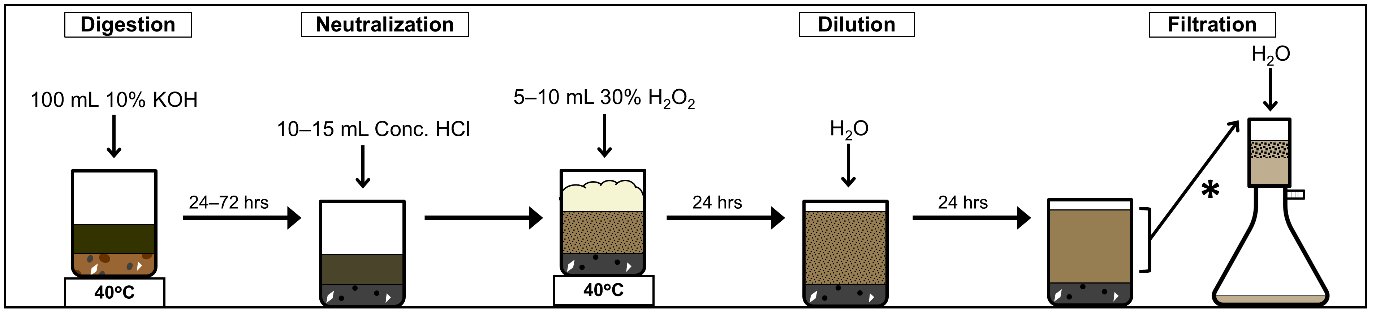


Figure S4. Schematic of method #4 where soils were digested using 10% KOH, neutralised using conc. HCl, and further digested using 30% H_2_O_2_.

*Oxidative digestion using NaClO*

Lastly, to enhance the removal of organic matter we applied oxidative digestion methods adapted from Enders et al. [8], Gouda et al. [9], and Monteiro et al. [10], using (i) NaClO (10–15% active chlorine) and (ii) a 1:1 ratio of NaClO:KOH.

For method #5, soil samples were treated with 150 mL of NaClO and then placed on a hotplate at 40°C (Fig. S5). A thin layer of bubbles formed at the top of the solution which subsided after 3–5 hours. A similar reaction was observed when the digest was kept at room temperature. The following day the soil particles settled to the bottom of the beaker and the solution appeared clear. The digest was then transferred to the SMI unit where it was made up to 700 mL with ultra-pure water and left overnight to settle. The following day a thick layer of organic matter consisting of undigested feathers and chitin floating formed at the top of the unit. Consequently, filtration was not always possible, and when filtration was possible, chitin covered the filter papers, making it challenging to see potential microplastic particles (Fig. S6). Similar results were produced when using a 30% 1:1 NaClO:KOH solution for method #6 (Fig. S7).


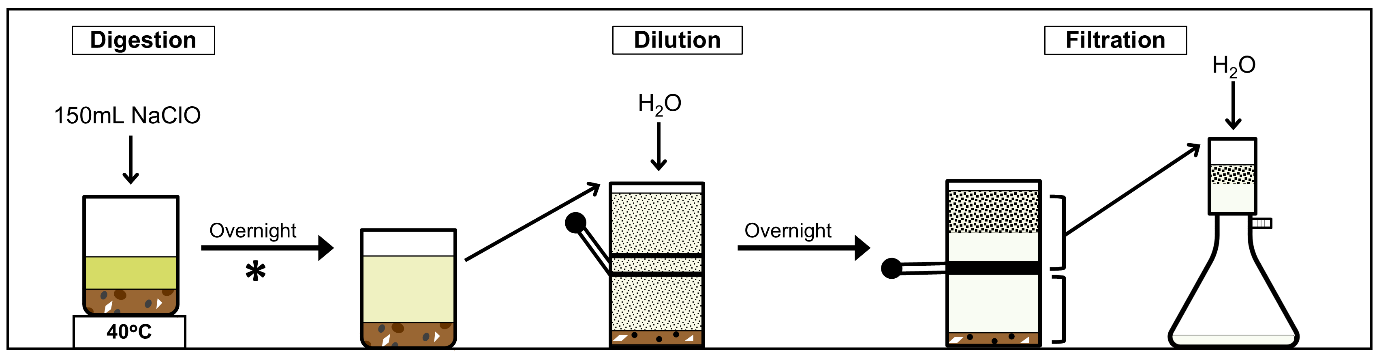


Figure S5. Schematic of method #5 where soils were digested using NaClO.


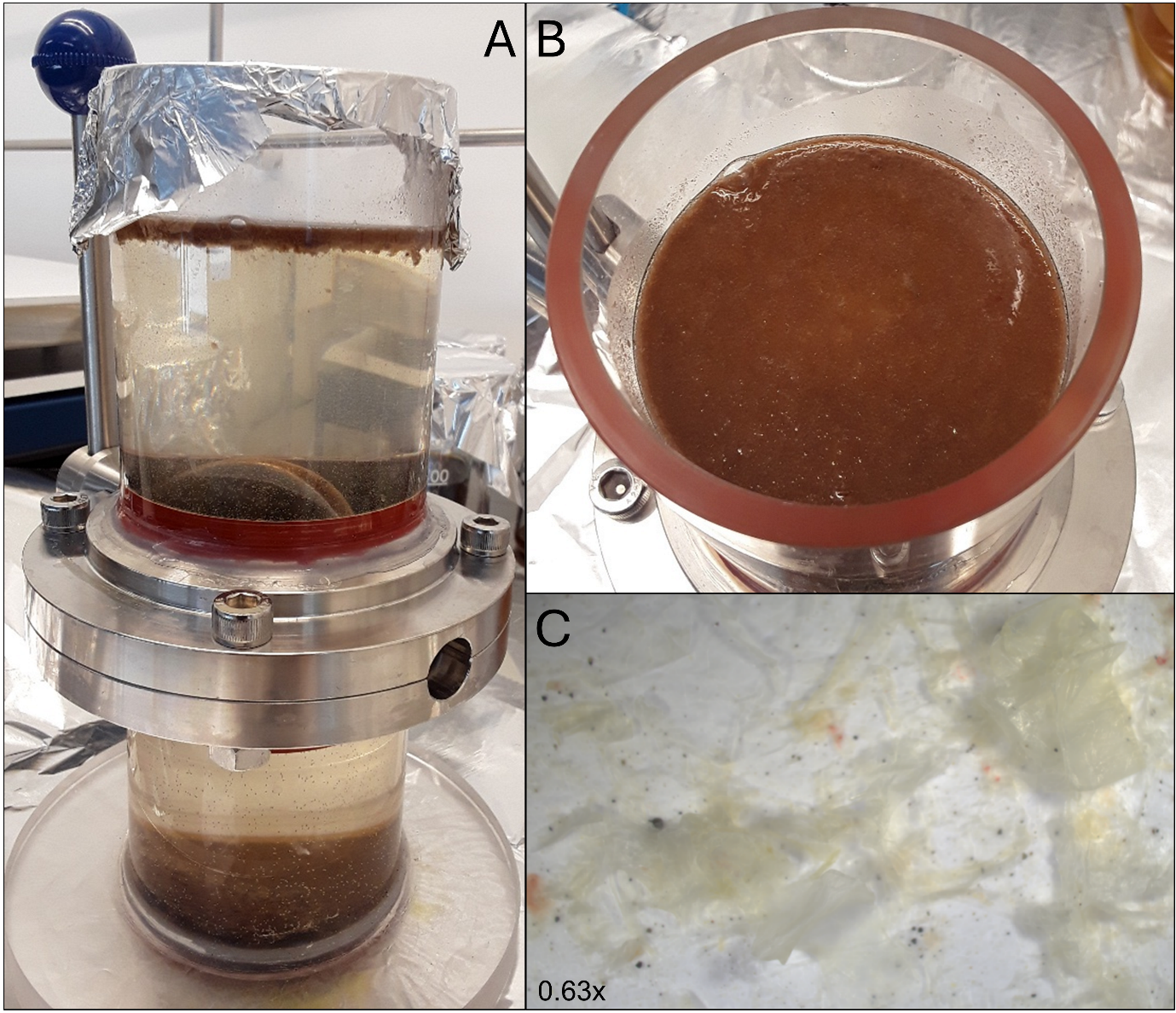


Figure S6. Images of samples after digestion steps using method #5 where (A & B) excessive amounts of chitin were present at the top of the SMI unit and (C) on the filter paper if filtration was possible.


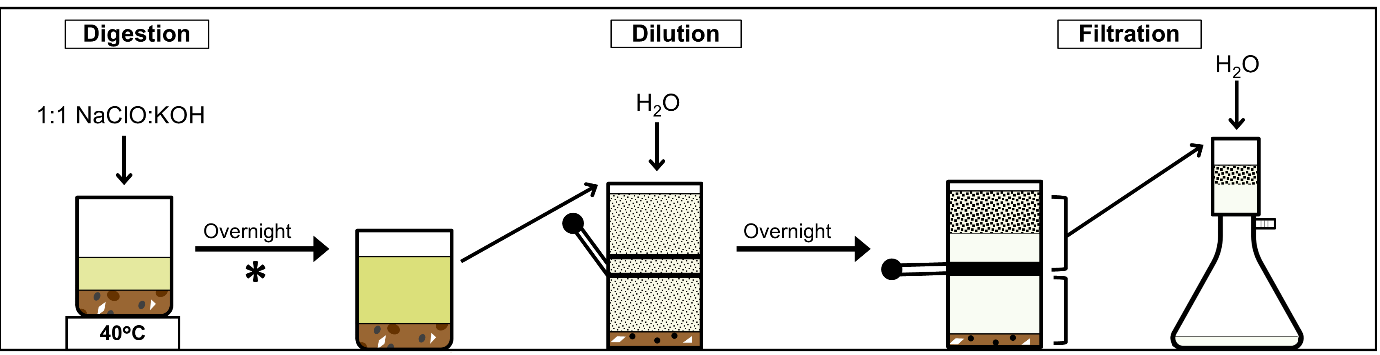


Figure S7. Schematic of method #6 where soils were digested using a 1:1 ratio of NaClO:KOH.

Appendix 2. Recovery rates (%) of reference polymers.

For method validation, one procedural blank and one 5 g (wet weight) ornithogenic soil sample were spiked with reference polymers. The method validation method was repeated three separate times, each with a different ornithogenic soil sample (CBA2-1, CBA1-1, CHA1-2). Reference polymers included one of each: polypropylene (PP), high-density polyethylene (HDPE), high impact polystyrene (HIPS), polyamide (PA), polyethylene terephthalate (PET), acrylonitrile butadiene styrene (ABS), and polyvinyl chloride (PVC) fragment (size: 1000–5000 μm); 10 polyethylene (PE) microbeads (100–500 μm), 10 polymethyl methacrylate (PMMA) fibres (1 mm; craft store), 10 HDPE fragments (500–1000 μm), and 10 PA fragments (500–1000 μm). The spiked method was carried out three times, each with a different ornithogenic soil sample, to ensure replicability.

**Trial #1:**

Spiked reagent blank:

| Standards: | Number inserted: | Number present: | | Recovery rate (%) |
| --- | --- | --- | --- | --- |
|  |  | Filter paper #1 | Filter paper #2 |  |
| PP (1000–5000 μm) | 1 | 1 |  | 1/1 (100%) |
| ABS (1000–5000 μm) | 1 |  | 1 | 1/1 (100%) |
| HDPE (1000–5000 μm) | 1 | 1 |  | 1/1 (100%) |
| HDPE (500–1000 μm) | 10 | 9 |  | 9/10 (90%) |
| PA (1000–5000 μm) | 1 |  | 1 | 1/1 (100%) |
| PA (500–1000 μm) | 10 |  | 7 | 7/10 (70%) |
| HIPS (1000–5000 μm) | 1 | 1 |  | 1/1 (100%) |
| PET (1000–5000 μm) | 1 |  | 1 | 1/1 (100%) |
| PMMA (1 mm) | 10 |  | 10 | 10/10 (100%) |
| PE (100–500 μm) | 10 | 10 |  | 10/10 (100%) |
| PVC (1000–5000 μm) | 1 |  | 1 | 1/1 (100%) |
| Total: | 47 | 22 | 21 | **(43/47) 91.48%** |

Spiked sample (CBA2-1):

| Standards: | Number inserted: | Number present: | | Recovery rate (%) |
| --- | --- | --- | --- | --- |
|  |  | Filter paper #1 | Filter paper #2 |  |
| PP (1000–5000 μm) | 1 | 1 |  | 1/1 (100%) |
| ABS (1000–5000 μm) | 1 |  | 1 | 1/1 (100%) |
| HDPE (1000–5000 μm) | 1 | 1 |  | 1/1 (100%) |
| HDPE (500–1000 μm) | 10 | 10 |  | 10/10 (100%) |
| PA (1000–5000 μm) | 1 |  | 1 | 1/1 (100%) |
| PA (500–1000 μm) | 10 |  | 10 | 10/10 (100%) |
| HIPS (1000–5000 μm) | 1 |  | 1 | 1/1 (100%) |
| PET (1000–5000 μm) | 1 |  | 1 | 1/1 (100%) |
| PMMA (1 mm) | 10 | 1 | 7 | 8/10 (80%) |
| PE (100–500 μm) | 10 | 10 |  | 10/10 (100%) |
| PVC (1000–5000 μm) | 1 |  | 1 | 1/1 (100%) |
| Total: | 47 | 23 | 22 | **45/47 (95.74%)** |

**Trial #2:**

Spiked reagent blank:

| Standards: | Number inserted: | Number present: | | Recovery rate (%) |
| --- | --- | --- | --- | --- |
|  |  | Filter paper #1 | Filter paper #2 |  |
| PP (1000–5000 μm) | 1 | 1 |  | 1/1 (100%) |
| ABS (1000–5000 μm) | 1 |  | 1 | 1/1 (100%) |
| HDPE (1000–5000 μm) | 1 | 1 |  | 1/1 (100%) |
| HDPE (500–1000 μm) | 10 | 8 |  | 8/10 (80%) |
| PA (1000–5000 μm) | 1 |  | 1 | 1/1 (100%) |
| PA (500–1000 μm) | 10 |  | 10 | 10/10 (100%) |
| HIPS (1000–5000 μm) | 1 |  | 1 | 1/1 (100%) |
| PET (1000–5000 μm) | 1 |  | 1 | 1/1 (100%) |
| PMMA (1 mm) | 10 |  | 8 | 8/10 (80%) |
| PE (100–500 μm) | 10 | 9 | 1 | 10/10 (100%) |
| PVC (1000–5000 μm) | 1 | 1 |  | 1/1 (100%) |
| Total: | 47 | 20 | 23 | **43/47 (91.48%)** |

Spiked sample (CBA1-1):

| Standards: | Number inserted: | Number present: | | Recovery rate (%) |
| --- | --- | --- | --- | --- |
|  |  | Filter paper #1 | Filter paper #2 |  |
| PP (1000–5000 μm) | 1 | 1 |  | 1/1 (100%) |
| ABS (1000–5000 μm) | 1 |  | 1 | 1/1 (100%) |
| HDPE (1000–5000 μm) | 1 | 1 |  | 1/1 (100%) |
| HDPE (500–1000 μm) | 10 | 10 |  | 10/10 (100%) |
| PA (1000–5000 μm) | 1 |  | 1 | 1/1 (100%) |
| PA (500–1000 μm) | 10 |  | 10 | 10/10 (100%) |
| HIPS (1000–5000 μm) | 1 |  | 1 | 1/1 (100%) |
| PET (1000–5000 μm) | 1 |  | 1 | 1/1 (100%) |
| PMMA (1 mm) | 10 |  | 10 | 10/10 (100%) |
| PE (100–500 μm) | 10 | 10 |  | 10/10 (100%) |
| PVC (1000–5000 μm) | 1 |  | 1 | 1/1 (100%) |
| Total: | 47 | 22 | 25 | **47/47 (100%)** |

**Trial #3:**

Spiked reagent blank:

| Standards: | Number inserted: | Number present: | | Recovery rate (%) |
| --- | --- | --- | --- | --- |
|  |  | Filter paper #1 | Filter paper #2 |  |
| PP (1000–5000 μm) | 1 | 1 |  | 1/1 (100%) |
| ABS (1000–5000 μm) | 1 |  | 1 | 1/1 (100%) |
| HDPE (1000–5000 μm) | 1 | 1 |  | 1/1 (100%) |
| HDPE (500–1000 μm) | 10 | 5 | 4 | 9/10 (90%) |
| PA (1000–5000 μm) | 1 |  | 1 | 1/1 (100%) |
| PA (500–1000 μm) | 10 |  | 10 | 10/10 (100%) |
| HIPS (1000–5000 μm) | 1 | 1 |  | 1/1 (100%) |
| PET (1000–5000 μm) | 1 |  | 1 | 1/1 (100%) |
| PMMA (1 mm) | 10 |  | 10 | 10/10 (100%) |
| PE (100–500 μm) | 10 | 9 | 1 | 9/10 (100%) |
| PVC (1000–5000 μm) | 1 |  | 1 | 1/1 (100%) |
| Total: | 47 | 17 | 29 | **46/47 (97.87%)** |

Spiked sample (CHA1-2):

| Standards: | Number inserted: | Number present: | | Recovery rate (%): |
| --- | --- | --- | --- | --- |
|  |  | Filter paper #1 | Filter paper #2 |  |
| PP (1000–5000 μm) | 1 | 1 |  | 1/1 (100%) |
| ABS (1000–5000 μm) | 1 |  | 1 | 1/1 (100%) |
| HDPE (1000–5000 μm) | 1 | 1 |  | 1/1 (100%) |
| HDPE (500–1000 μm) | 10 | 10 |  | 10/10 (100%) |
| PA (1000–5000 μm) | 1 |  | 1 | 1/1 (100%) |
| PA (500–1000 μm) | 10 |  | 10 | 10/10 (100%) |
| HIPS (1000–5000 μm) | 1 |  | 1 | 1/1 (100%) |
| PET (1000–5000 μm) | 1 |  | 1 | 1/1 (100%) |
| PMMA (1 mm) | 10 |  | 10 | 10/10 (100%) |
| PE (100–500 μm) | 10 |  | 10 | 10/10 (100%) |
| PVC (1000–5000 μm) | 1 |  | 1 | 1/1 (100%) |
| Total: | 47 | 12 | 35 | **47/47 (100%)** |

Appendix 3. Reference polymer spectra

Reference polymer spectra before (black) and after (blue) microplastic extraction methods.

Polypropylene (PP) fragment:

**
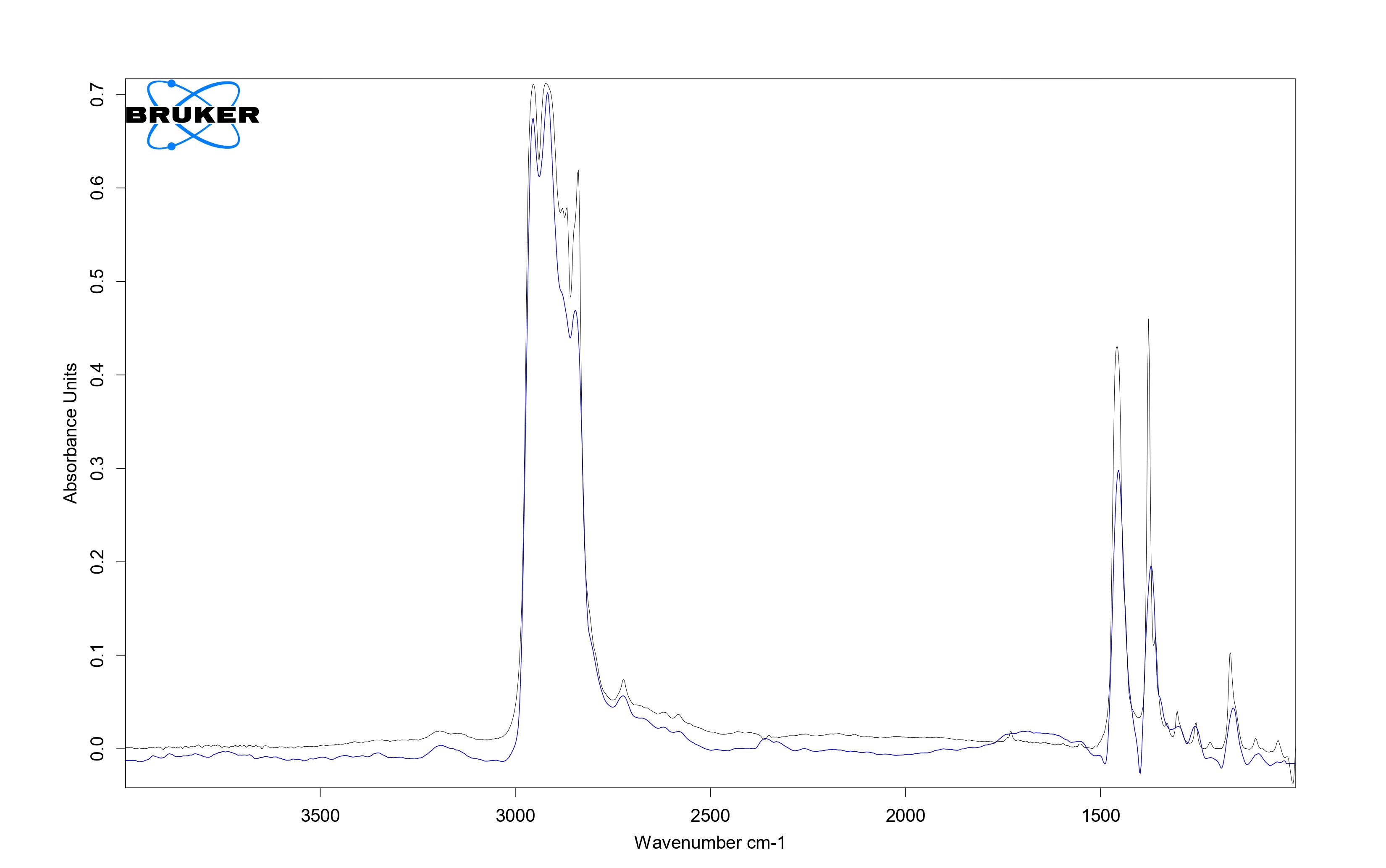
**

High-density polyethylene (HDPE) fragment:


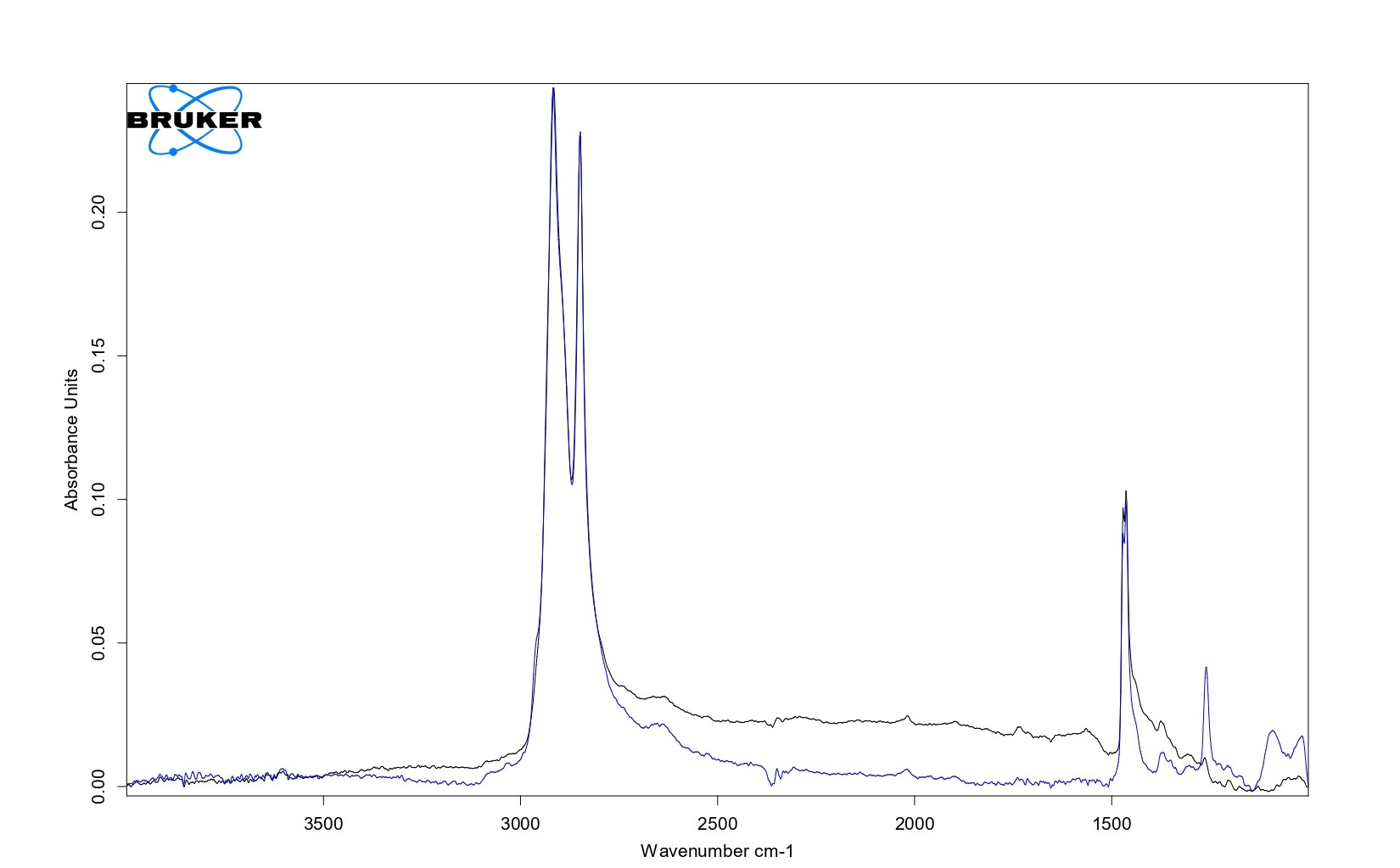


High impact polystyrene (HIPS) fragment:


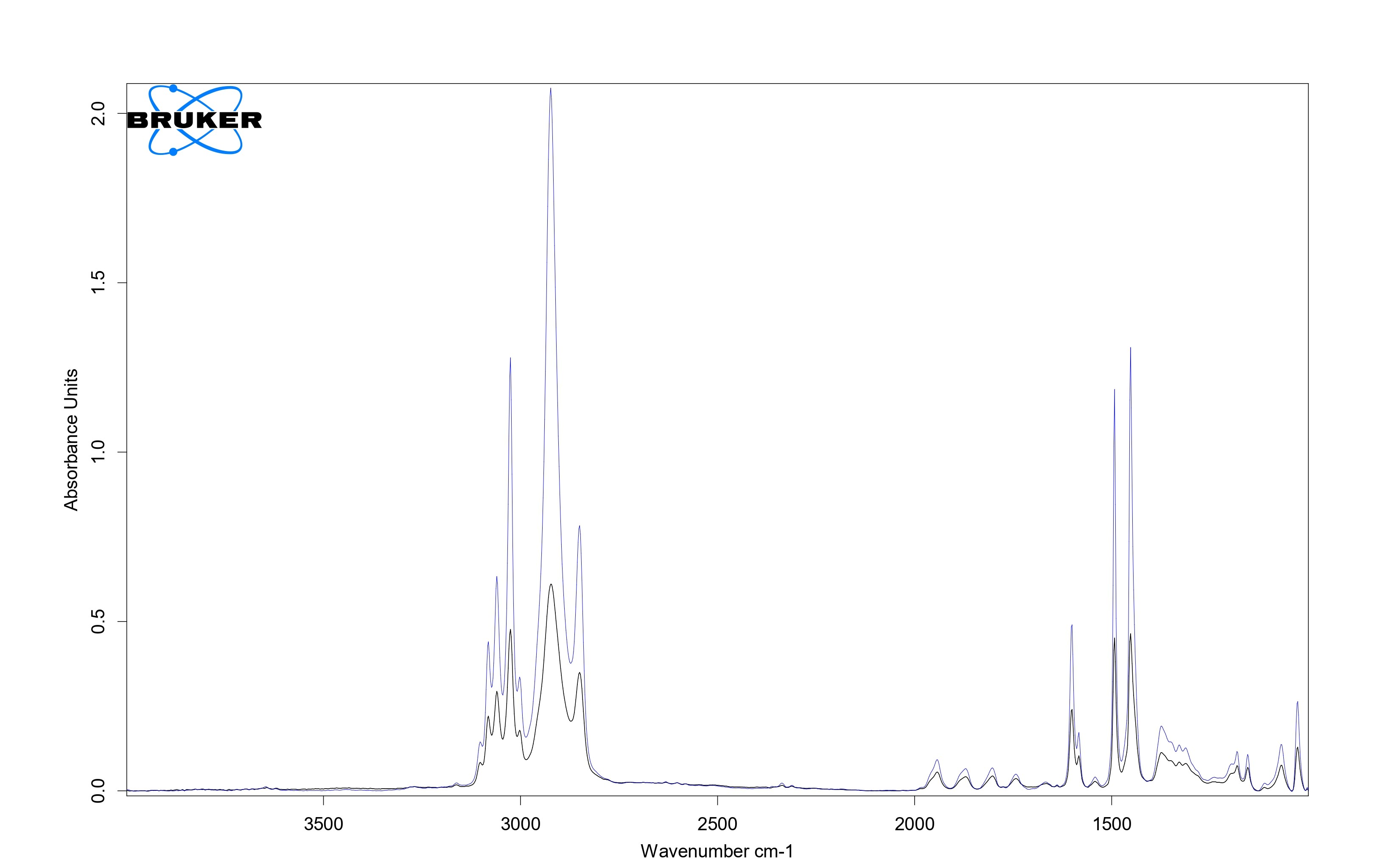


Polyamide (PA) fragment:


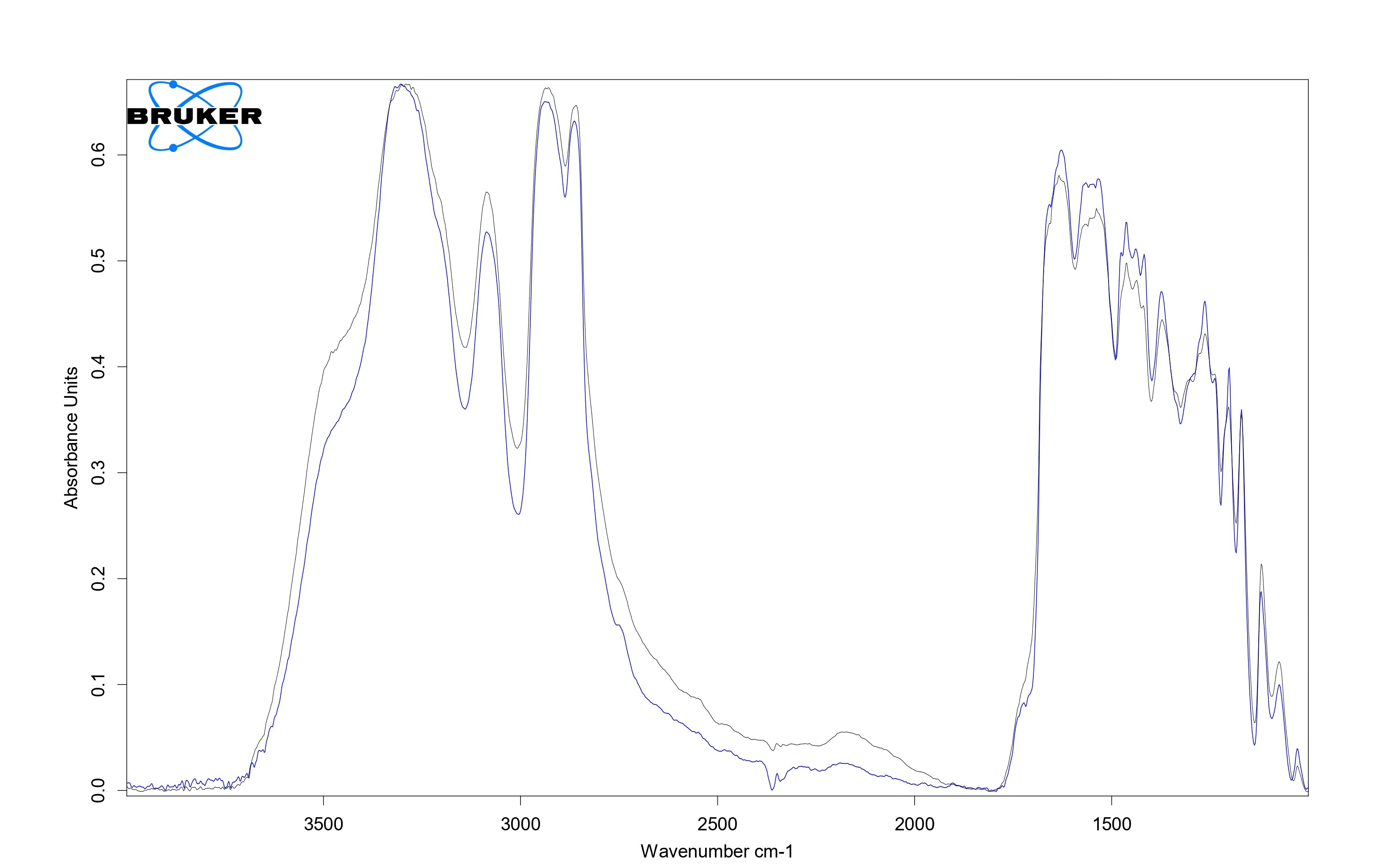


Polyethylene terephthalate (PET) fragment:


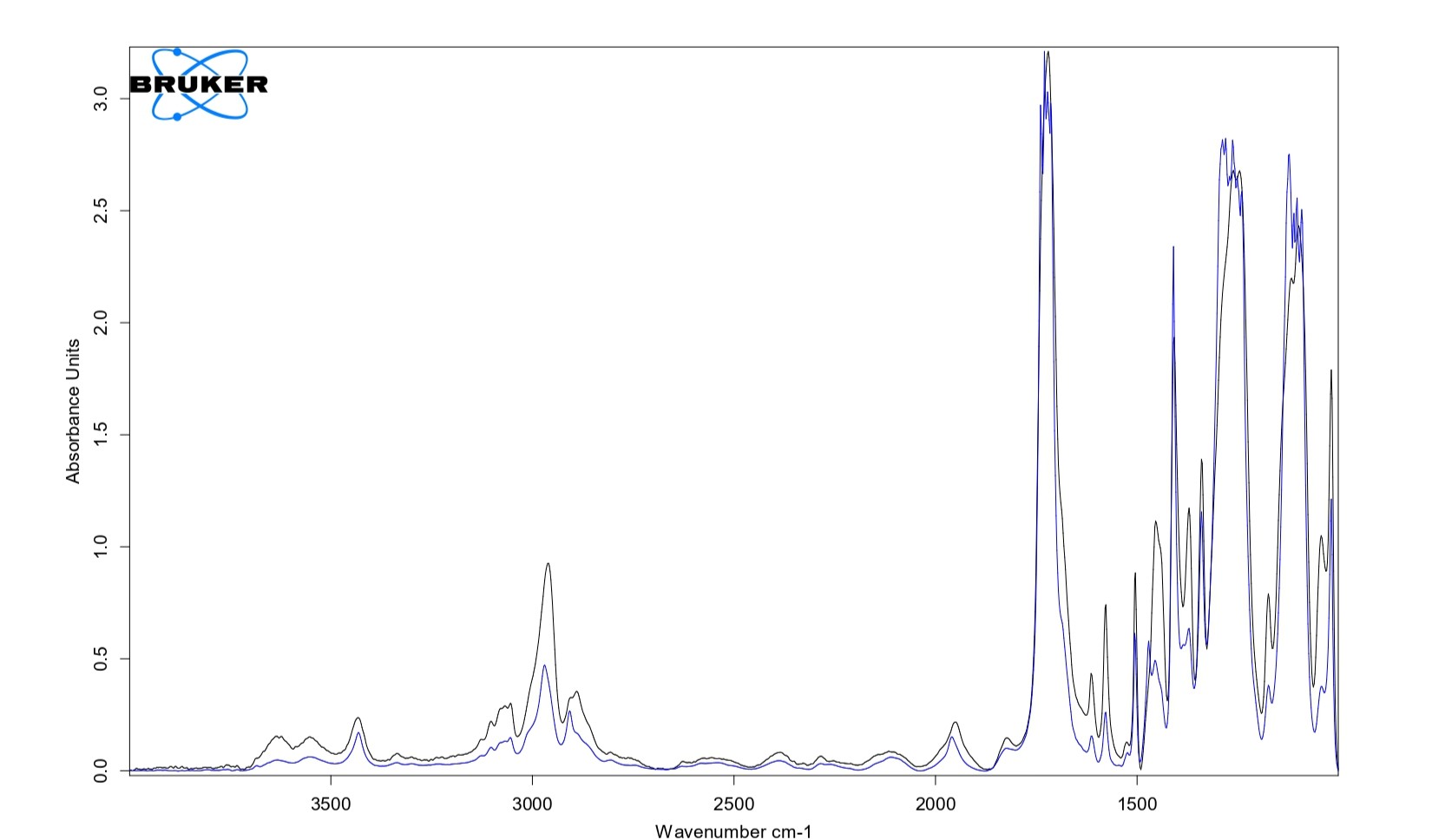


Acrylonitrile butadiene styrene (ABS) fragment:


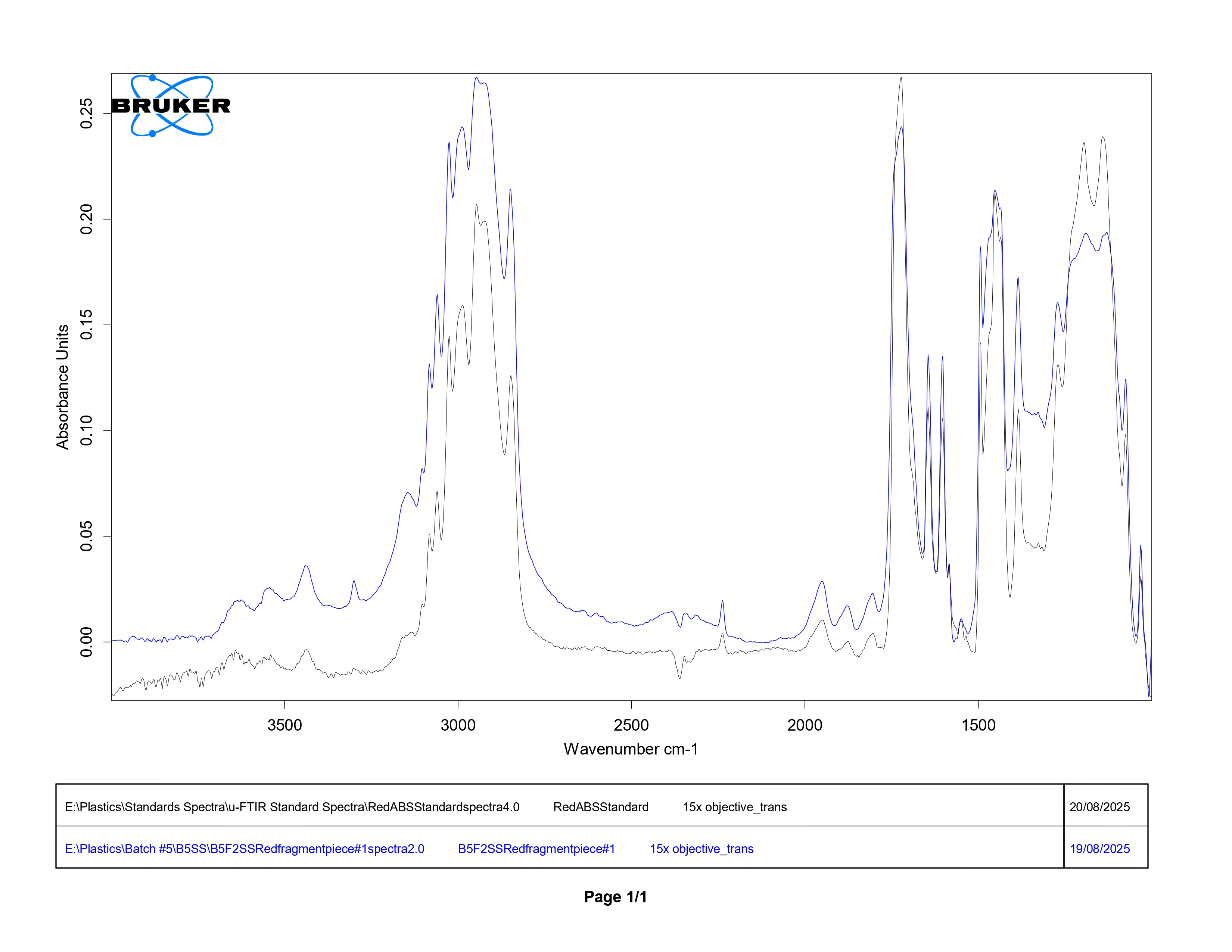


Polyvinyl chloride (PVC) fragment:


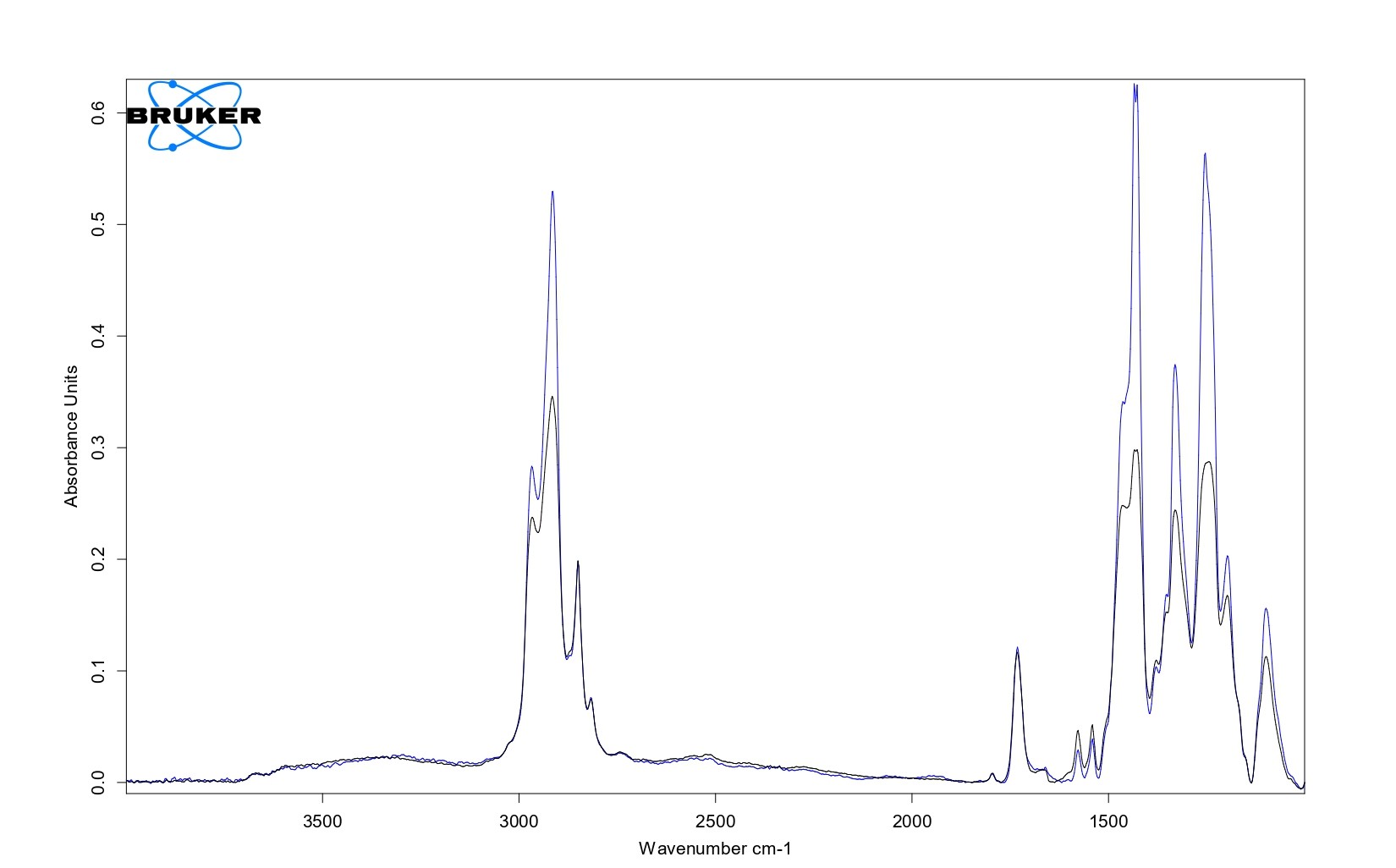


Polyethylene (PE) microbead:


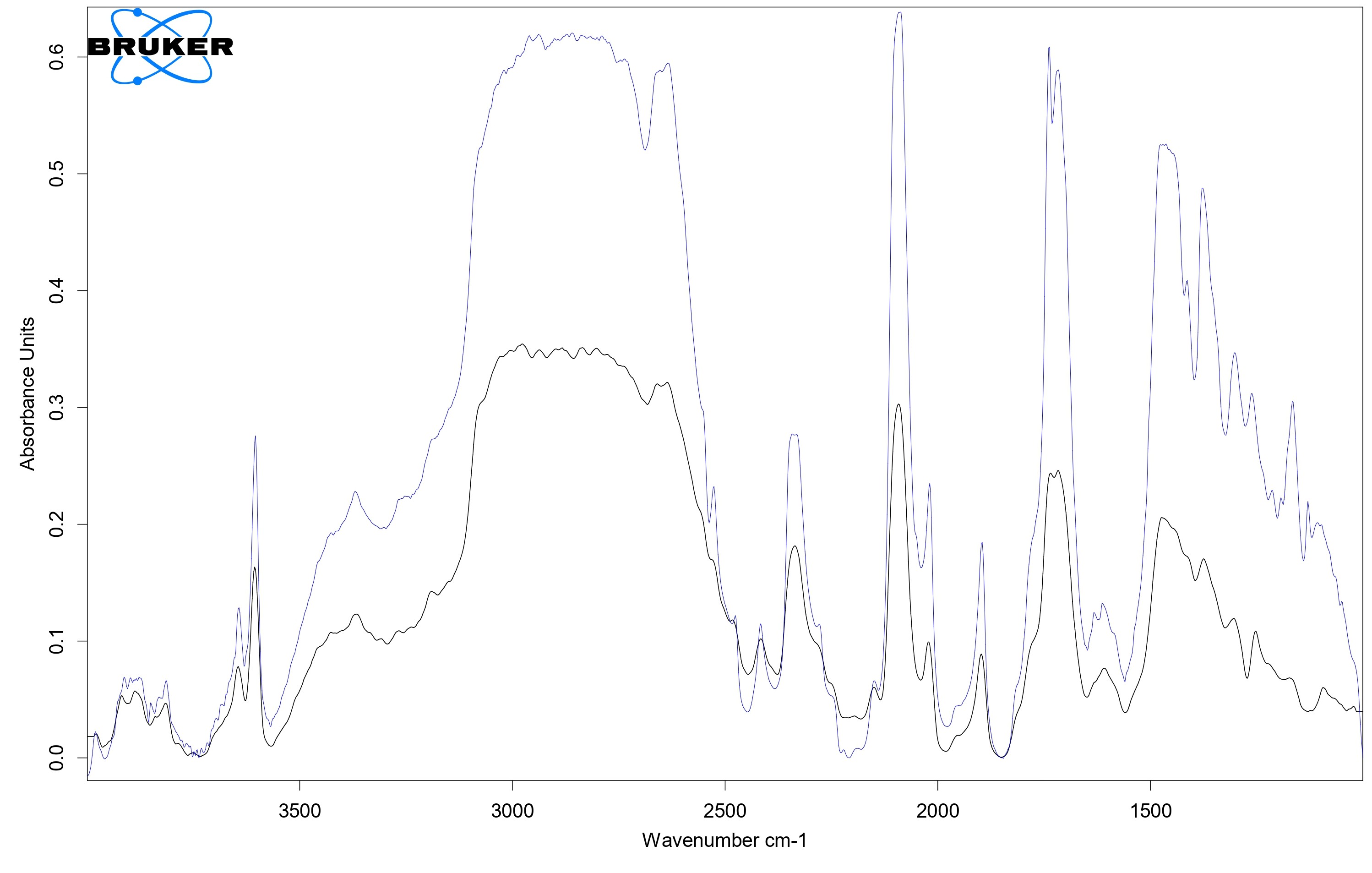


Polymethyl methacrylate (PMMA) fibre:


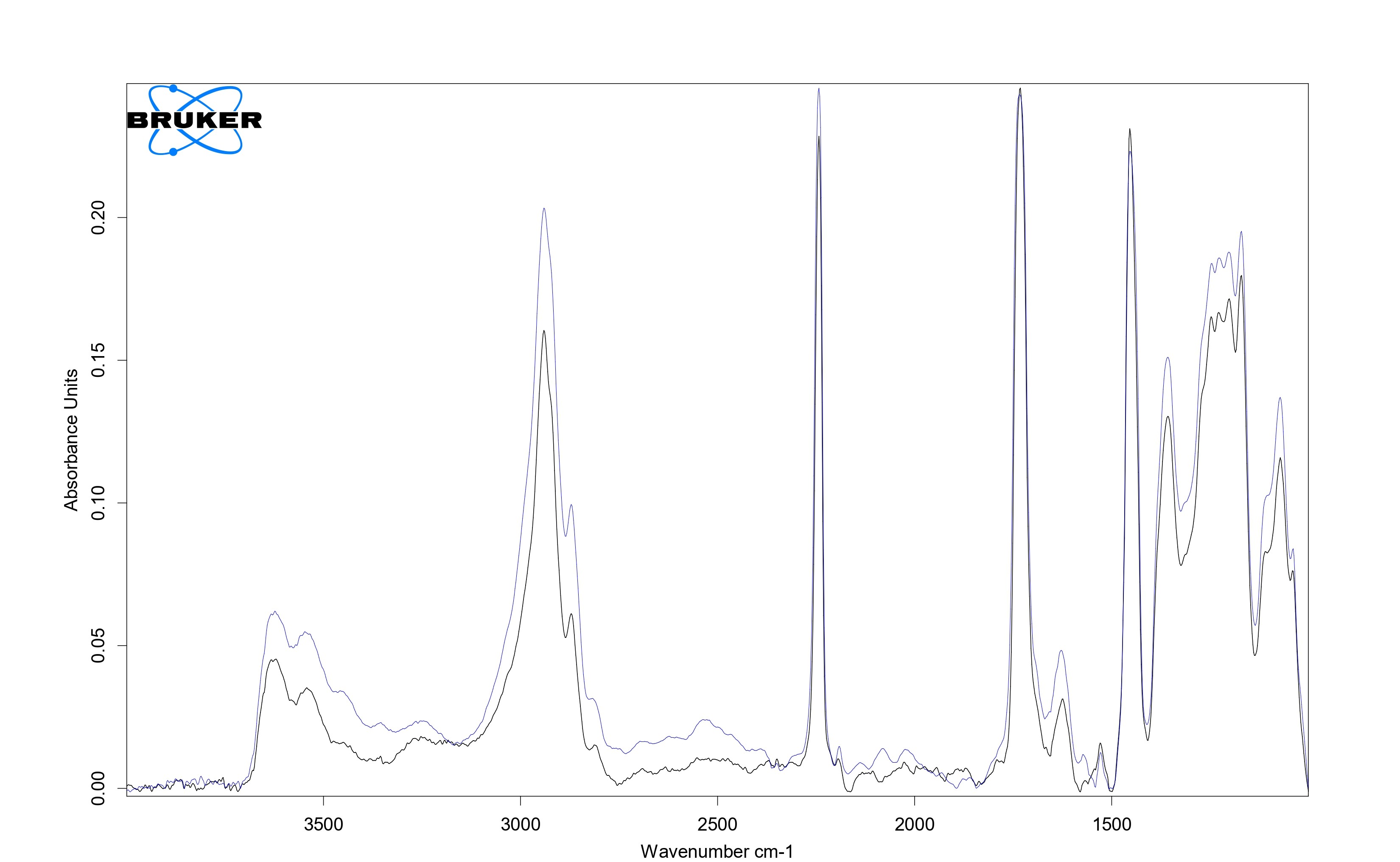


**References**

[1] R.R. Hurley, A.L. Lusher, M. Olsen, L. Nizzetto, Validation of a Method for Extracting Microplastics from Complex, Organic-Rich, Environmental Matrices, Environ. Sci. Technol. 52 (2018) 7409–7417. https://doi.org/10.1021/acs.est.8b01517.

[2] H. Ruffell, O. Pantos, B. Robinson, S. Gaw, A method for the extraction of microplastics from solid biowastes including biosolids, compost, and soil for analysis by µ-FTIR, MethodsX 12 (2024) 102761. https://doi.org/10.1016/j.mex.2024.102761.

[3] F. Bessa, N. Ratcliffe, V. Otero, P. Sobral, J.C. Marques, C.M. Waluda, P.N. Trathan, J.C. Xavier, Microplastics in gentoo penguins from the Antarctic region, Sci Rep 9 (2019) 14191. https://doi.org/10.1038/s41598-019-50621-2.

[4] J.C. Prata, J.P. Da Costa, A.V. Girão, I. Lopes, A.C. Duarte, T. Rocha-Santos, Identifying a quick and efficient method of removing organic matter without damaging microplastic samples, Science of The Total Environment 686 (2019) 131–139. https://doi.org/10.1016/j.scitotenv.2019.05.456.

[5] A. Karami, A. Golieskardi, C.K. Choo, N. Romano, Y.B. Ho, B. Salamatinia, A high-performance protocol for extraction of microplastics in fish, Science of The Total Environment 578 (2017) 485–494. https://doi.org/10.1016/j.scitotenv.2016.10.213.

[6] M.B. Alfonso, K. Takashima, S. Yamaguchi, M. Tanaka, A. Isobe, Microplastics on plankton samples: Multiple digestion techniques assessment based on weight, size, and FTIR spectroscopy analyses, Marine Pollution Bulletin 173 (2021) 113027. https://doi.org/10.1016/j.marpolbul.2021.113027.

[7] A.L. Dawson, C.A. Motti, F.J. Kroon, Solving a Sticky Situation: Microplastic Analysis of Lipid-Rich Tissue, Front. Environ. Sci. 8 (2020) 563565. https://doi.org/10.3389/fenvs.2020.563565.

[8] K. Enders, R. Lenz, S. Beer, C.A. Stedmon, Extraction of microplastic from biota: recommended acidic digestion destroys common plastic polymers, ICES Journal of Marine Science 74 (2017) 326–331. https://doi.org/10.1093/icesjms/fsw173.

[9] M.Z. Gouda, S. Roberge, L. Khiari, R. Benjannet, M. Desrosiers, Novel integrated workflow for microplastics extraction, quantification, and characterization in organic fertilizing residuals using micro-Fourier transform infrared spectroscopy (μ-FTIR), Chemosphere 377 (2025) 144357. https://doi.org/10.1016/j.chemosphere.2025.144357.

[10] S.S. Monteiro, T. Rocha-Santos, J.C. Prata, A.C. Duarte, A.V. Girão, P. Lopes, T. Cristovão, J.P. Da Costa, A straightforward method for microplastic extraction from organic-rich freshwater samples, Science of The Total Environment 815 (2022) 152941. https://doi.org/10.1016/j.scitotenv.2022.152941.
